# A radiological visual scale to predict the potentially recruitable lung in ALI/ARDS patients

**DOI:** 10.1186/cc9625

**Published:** 2011-03-11

**Authors:** D Chiumello, M Cressoni, A Marino, E Gallazzi, M Brioni, MC Andrisani, M Lazzerini, P Biondetti

**Affiliations:** 1 Fondazione IRCCS Ca' Granda-Ospedale Maggiore Policlinico, Milan, Italy; 2 Università degli Studi di Milano, Milan, Italy

## Introduction

In ALI/ARDS patients the amount of potentially recruitable lung is extremely variable and it is poorly predictable by the changes of oxygenation, carbon dioxide or compliance during a PEEP trial [1]. At the present time the gold standard to compute the lung recruitability is the quantitative lung CT scan, in which each lung image, after being manually drawn, is analyzed by dedicated software. However, this is both a laborious and time-consuming technique. The aim of this study was to evaluate the ability of a visual radiological scale compared with lung CT scan analysis to predict the lung recruitability in ALI/ARDS patients.

## Methods

A whole lung CT scan was performed at 5 and 45 cmH_2_O airway pressure. For CT scan analysis each lung image was manually outlined and analyzed by a dedicated software. The potentially recruitable lung was defined as the proportion of the nonaerated lung tissue in which aeration was restored [1]. For radiological visual scale analysis, two radiologists performed a blinded evaluation of the consolidation/collapsed areas in each lobe by visual inspection [2]. The overall lung change in consolidation/collapsed was obtained by the sum of each lobe and computed as the difference between the two conditions.

## Results

Twenty-four ALI/ARDS patients (age 59 ± 15 years, BMI 26 ± 4 kg/m^2^, PaO_2_/FiO_2 _170 ± 60, PEEP 10 ± 2 cmH_2_O) were enrolled. The percentage of potentially recruitable lung was 16.2 ± 7.1% and 14.7 ± 7.0%, computed by CT scan and by the visual radiological scale, respectively. The mean difference between CT scan analysis and visual radiological analysis was 3.3 ± 4.6% (median: 2.91, interquartile range: 0.38 to 6.56). The error of the visual method was lower than 5% in 14 patients (58.3%), between 5% and 10% in eight patients (33.3%) and between 10% and 15% in two patients (8.3%).

## Conclusions

The application of a radiological visual scale is able to predict the amount of potentially recruitable lung similarly to those obtained by a dedicated software avoiding the need of manually drawing each lung image.
